# Born with two faces: sequential DLBCL, NOS and TFHL-AI with *TET2* mutation – a case report

**DOI:** 10.3389/fimmu.2026.1698958

**Published:** 2026-02-13

**Authors:** Qing Li, Shishuo Dai, Chenlu Yang, Weiping Liu, Yu Wu

**Affiliations:** 1 Department of Hematology and Institute of Hematology, West China Hospital, Sichuan University, Chengdu, Sichuan, China; 2 Department of Pathology, West China Hospital, Sichuan University, Chengdu, Sichuan, China

**Keywords:** TET2 mutation, next-generation sequencing, diffuse large B-cell lymphoma, not otherwise specified, nodal T follicular helper cell lymphoma, angioimmunoblastic type, case report

## Abstract

Diffuse large B-cell lymphoma, not otherwise specified (DLBCL, NOS) and nodal T follicular helper cell lymphoma, angioimmunoblastic type (TFHL-AI) share significant histopathological and pathogenetic similarities. However, the mechanisms underlying these overlaps remain insufficiently explored in the literature. We report the case of a 74-year-old man who initially presented with progressive sore throat and was diagnosed with DLBCL, NOS based on a tonsillar biopsy. He achieved complete remission following six cycles of R-CHOP chemotherapy (rituximab, cyclophosphamide, vindesine, liposomal doxorubicin, and dexamethasone). However, the patient was lost to follow-up. About two years later, he re-presented with generalized pruritus and lymphadenopathy. A cervical lymph node biopsy confirmed TFHL-AI. He received four cycles of the histone deacetylase inhibitor (HDACi) chidamide combined with COEP chemotherapy (cyclophosphamide, vindesine, etoposide, and prednisone), resulting in a partial remission. However, the disease subsequently progressed, and the patient passed away six months later, with a total overall survival of 35months. Next-generation sequencing (NGS) of biopsy specimens from both lymphoma types revealed shared *TET2* mutations. These findings suggest that *TET2* mutations may drive clonal evolution and reprogram the tumor microenvironment, potentially facilitating divergent evolution from a common mutated precursor or the sequential development of distinct lymphoid neoplasms. This case highlights the diagnostic and therapeutic challenges of TFHL-AI following DLBCL, NOS. Although the prognosis is generally poor, treatment combining HDAC inhibitors such as chidamide with chemotherapy may offer therapeutic potential. Further studies are needed to clarify the molecular mechanisms underlying such lymphoid evolution and to guide optimal management.

## Introduction

Diffuse large B-cell lymphoma, not otherwise specified (DLBCL, NOS) is a lymphoid malignancy in adults characterized by significant clinical and genetic heterogeneity (1). Nodal T follicular helper cell lymphoma, angioimmunoblastic type (TFHL-AI), a subtype of mature T-cell lymphoma, is associated with poor prognosis. Key mutations frequently observed in TFHL-AI include *RHO*A, *TET2*, *DNMT3A*, and *IDH2* (2). Although cases of DLBCL, NOS developing after TFHL-AI, and vice versa, have been reported (3, 4), this study performed deep sequencing of biopsy tissue samples from two disease states to explore the potential links between DLBCL, NOS and TFHL-AI.

## Case description

In August 2018, a 74-year-old man developed a sore throat that progressively worsened. However, he did not seek medical attention at Sichuan Cancer Hospital until December 2018. Prior to this, his sore throat persisted despite symptomatic treatment at a community hospital. The patient denied fever, night sweats, or weight loss, and had no significant medical history or family history of genetic diseases. Physical examination revealed bilateral tonsillar enlargement. In December 2018, a tonsil biopsy was performed and sent to the Department of Pathology at West China Hospital, Sichuan University, for further analysis.

The patient’s complete blood count, coagulation markers, liver and kidney function parameters, electrolytes, cardiac ultrasound, and electrocardiography were all within normal limits. Serum Epstein–Barr virus (EBV) DNA level was 6.29 × 10^1^ copies/mL. Positron emission tomography/computed tomography (PET/CT) revealed abnormal FDG uptake in multiple regions: bilateral oropharyngeal lateral walls, left posterior nasopharyngeal wall, bilateral submandibular glands, bilateral cervical paravascular regions, and left posterior cervical triangle. The Deauville score was 5. A diagnosis of DLBCL, NOS was established. From December 2018 to April 2019, the patient underwent six cycles of R-CHOP chemotherapy (rituximab, cyclophosphamide, vindesine, liposomal doxorubicin, and dexamethasone), achieving complete remission as confirmed by PET/CT (Deauville score 2). However, the patient was not followed up regularly thereafter.

**Figure 1:**
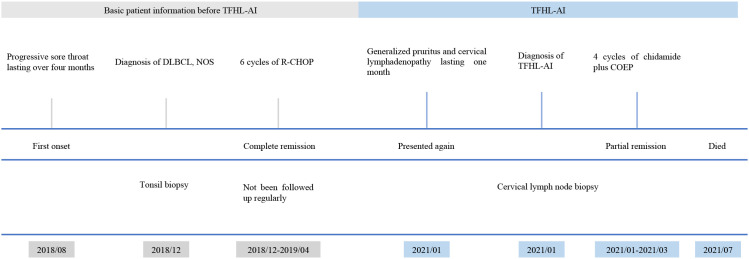
A summary of the patient’s clinical course.

In January 2021, about two years after being diagnosed with DLBCL, the patient developed systemic itching and cervical lymph node enlargement that lasted for one month. A physical examination revealed palpable cervical lymph nodes, and a biopsy confirmed TFHL-AI. PET/CT (positron emission tomography/computed tomography) showed hypermetabolic lymph nodes in the cervical, thoracic, and abdominal regions, while bone marrow biopsy was negative. Serum EBV DNA was elevated (1.25 × 10^4^ copies/mL). Given that the patient was diagnosed with post-DLBCL TFHL-AI, NOS and extensive disease, the patient received four courses of treatment with chidamide combined with COEP regimen (cyclophosphamide, vincristine, etoposide and prednisone) from January to March 2021. After 4 cycles, the patient achieved partial remission, with EBV DNA decreasing to 2.30 × 10² copies/mL. However, due to disease progression, the patient died in July 2021, 6 months after being diagnosed with TFHL-AI, with an overall survival of 35 months. A summary of the clinical course is shown in **Figure 1**.

**Figure 2:**
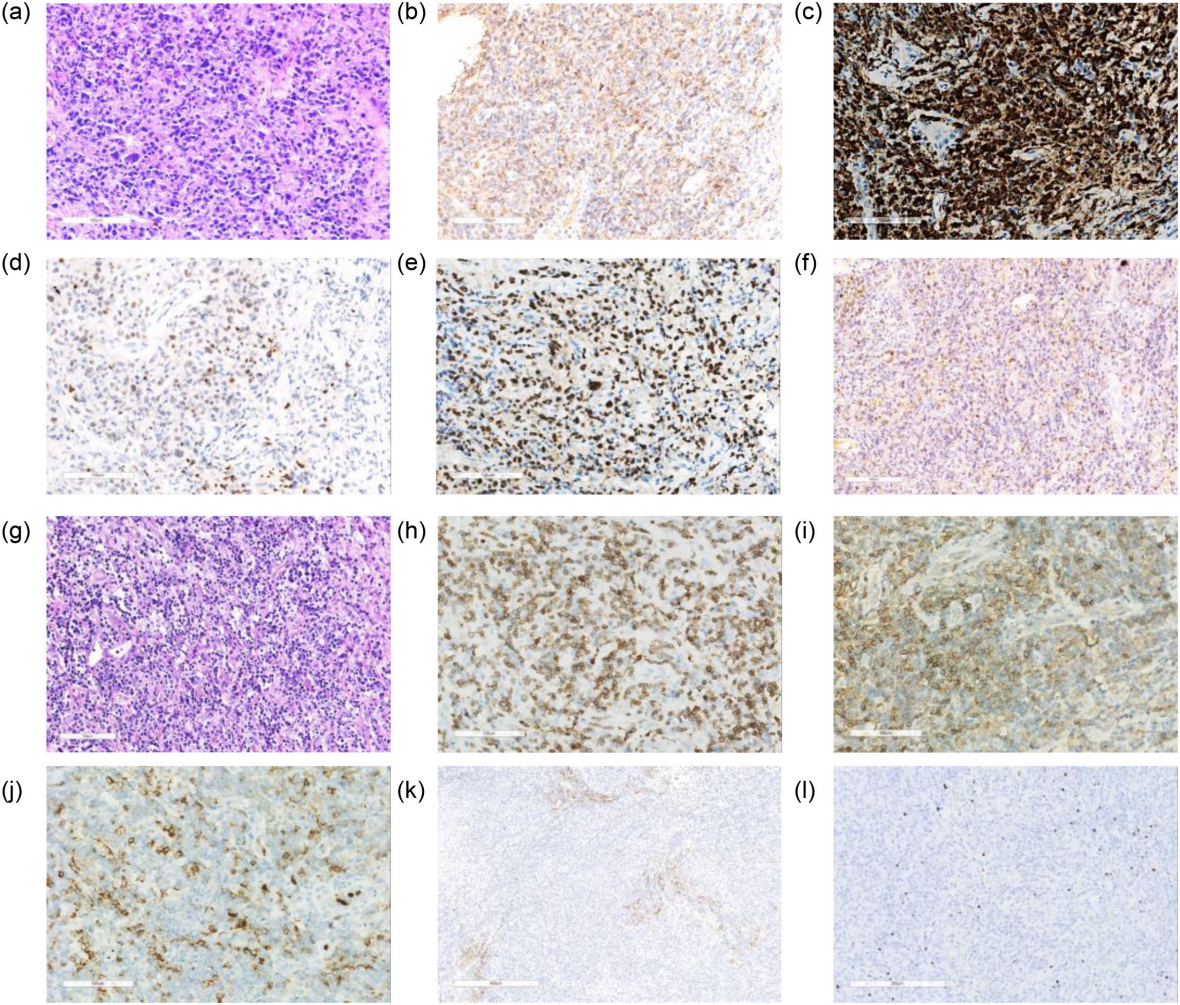
Histopathological, immunohistochemical, and ISH findings at DLBCL, NOS and TFHL-AI **(a–f) (a)** Hematoxylin and eosin (H&E) staining shows diffuse infiltration of large atypical lymphoid cells with vesicular nuclei, prominent nucleoli, and focal areas of necrosis (original magnification ×100); **(b)** Positive for CD20; **(c)** Positive for CD79a; **(d)** Positive for MUM-1; **(e)** Positive for Ki-67 (MIB-1, ~80%); **f** ISH for EBER1/2 reveals strong nuclear positivity in tumor cells, confirming EBV association (EBER-ISH, ×100, +, ~20%). All images were taken from the same tonsillar biopsy specimen. **(g–l)** Histopathological, immunohistochemical, and ISH findings at diagnosis of TFHL-AI **(g)** Hematoxylin and eosin staining showed partially naked follicles, expanded interfollicular zones, and prominent vascular proliferation; **(h)** Positive for CD3; **(i)** Positive for CD4; **(j)** Partial positivity for CD10; **(k)** CD21 highlights follicular dendritic cell meshworks; **(l)** EBER1/2 ISH showing nuclear positivity in scattered lymphocytes.

Tonsil biopsy revealed diffuse infiltration of large atypical lymphoid cells with focal necrosis (**Figure 2a**). Immunohistochemical staining showed that the atypical cells were positive for CD20 (**Figure 2b**), CD79a (**Figure 2c**), MUM-1 (**Figure 2d**), BCL-6 (data not shown), and Ki-67 (MIB-1, ~80%; **Figure 2e**). EBV infection was confirmed by EBER1/2 ISH (~20%; **Figure 2f**). The cells were negative for CD30, CD56, GB, TIA-1, and BCL-2. Although approximately 20% of EBER-positive tumor cells were detected, this case was not suitable for diagnosis as EBV-positive DLBCL, NOS according to the WHO-HAEM5 and ICC (2022) criteria (requiring >80% EBER-positive tumor cells) (5). Therefore, we diagnosed this case as DLBCL, NOS. Gene rearrangement analysis demonstrated monoclonal IGK and IGH rearrangements. According to the Hans algorithm, the DLBCL was classified as non-germinal center B-cell-like (non-GCB) subtype.

Cervical lymph node biopsy showed partially naked follicles, expanded interfollicular zones, and prominent vascular proliferation (**Figure 2g**). Tumor cells were positive for CD3, CD4, and partially for CD10 (**Figures 2h–j**). CD21 highlighted follicular dendritic cell meshworks (**Figure 2k**). EBER1/2 ISH revealed dispersed nuclear positivity, mainly in small lymphocytes with occasional large positive cells (**Figure 2l**). Ki-67 was ~60%. Tumor cells were negative for CD20, CXCL13, CD8, and CD15. They expressed PD-1, CD43, and CD30 (restricted to interfollicular large cells). PAX-5 showed weak focal staining (images not shown). Clonal TCRγ rearrangement was detected. No evidence of monoclonal IGH rearrangement was detected. No RHOA (G17V) or IDH2 (R172) mutations were found by Sanger sequencing.

**Figure 3:**
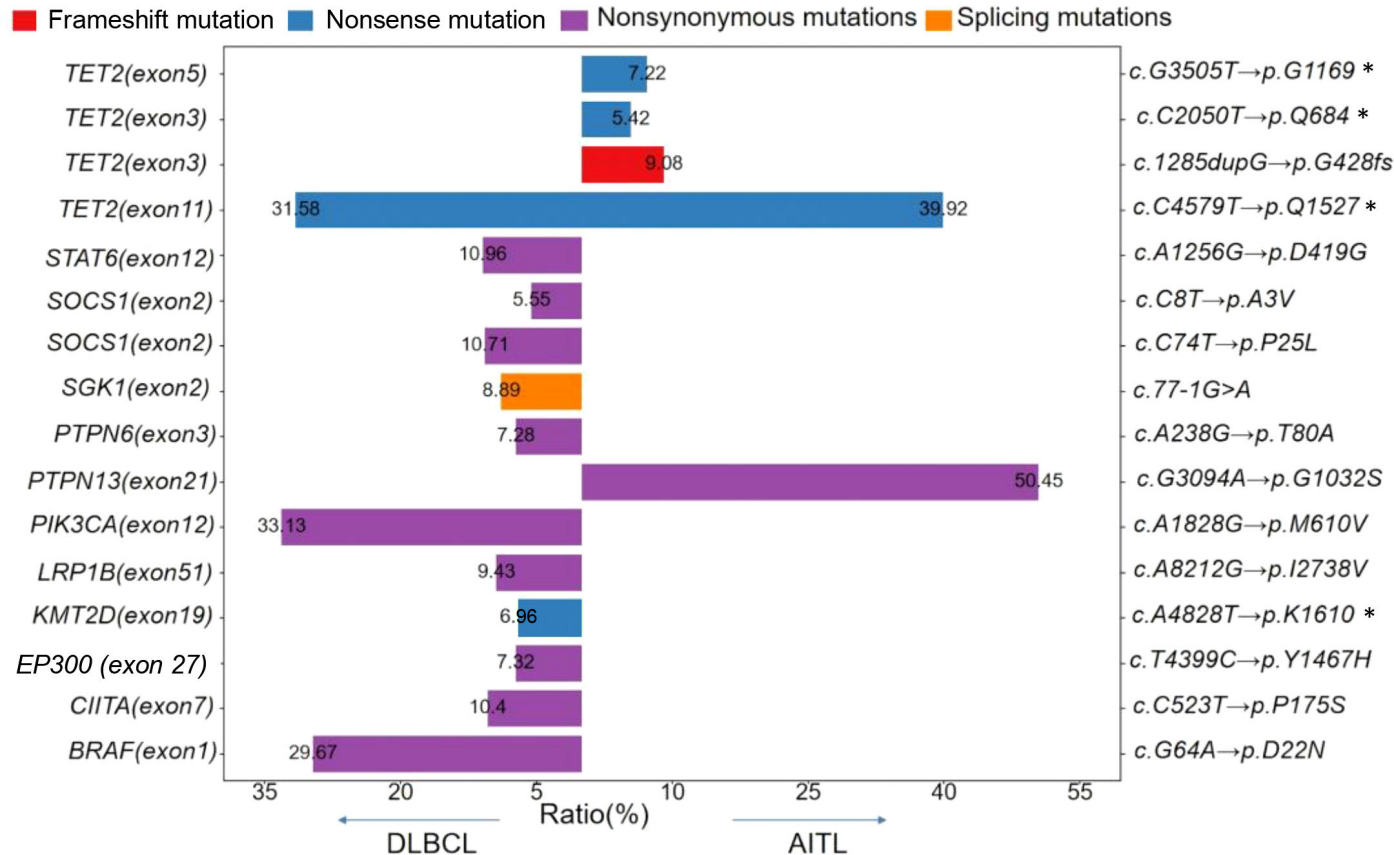
Mutational landscape of our reported case at the time of diagnosis of DLBCL, NOS and TFHL-AI The color represents the type of mutation, the left ordinate represents the mutation gene and mutation site, and the right vertical coordinate represents the change of nucleotides and amino acids. The abscissa represents the frequency of mutations. Genes covered by both sequencing panels include *TET2*, *SOCS1*, *LRP1B*, *KMT2D*, *EP300*, and *CIITA*. Mutations in genes not shared between panels—such as *STAT6*, *SGK1*, *PTPN6*, *PIK3CA*, *BRAF* (DLBCL only) and *PTPN13* (AITL only)—are not directly comparable.

At the time of DLBCL, NOS diagnosis, a *TET2* nonsense mutation, c.C4579T (p.Q1527*), was identified by next-generation sequencing (NGS) in a tonsillar specimen. The same mutation was later detected in a lymph node specimen obtained at the time of TFHL-AI diagnosis. Notably, three additional *TET2* variants with lower variant allele frequencies (VAFs) were identified exclusively in the TFHL-AI sample. Among the genes covered by both sequencing panels, *TET2* was found to be mutated in both lymphoma types. The VAF of the shared *TET2* mutation was 31.58% in the DLBCL, NOS sample and 39.92% in the TFHL-AI sample (**Figure 3**).

## Discussion

While *TET2* mutations are rare in DLBCL, NOS, they are more common in TFHL-AI. The role of *TET2* mutations in the progression from DLBCL to TFHL-AI remains unclear and requires further research. TFHL-AI may develop from age-related clonal hematopoiesis (ACH), where *TET2* mutations in ACH-derived germinal center B cells induce clonal evolution. These mutated B cells can function as microenvironmental cells, promoting TFHL-AI tumorigenesis (6). Studies indicate that 10%–60% of polyclonal B cells in TFHL-AI lymph nodes harbor the same *TET2* mutation found in corresponding T-cell lymphoma clones (7). Furthermore, *TET2*-mutated premalignant cells can differentiate into both T-lineage tumor cells and B cells (7, 8). Determining the role of *TET2* in epigenetic regulation is crucial for understanding the complex, multilineage tumorigenesis of TFHL-AI and offers new directions for research and therapeutic strategies (9).

In this context, our case likely reflects the divergent evolution of a mutated hematopoietic precursor into both B- and T-cell lymphoproliferative disorders. This paradigm has been previously described in TFHL-AI and associated myeloid neoplasms, and is further supported by recent studies such as Lewis et al. (10), which demonstrated a molecular and clonal link between TFHL-AI and B-cell proliferations. While DLBCL, NOS with secondary TFHL-AI is rarely reported, B-cell LPDs in the context of TFHL-AI are increasingly recognized, supporting a shared clonal origin and parallel evolution driven by TET2-mutated B cells within a TFH-rich microenvironment.

Although the initial biopsy did not meet the criteria for TFHL-AI, the presence of an underlying TFH-cell clone cannot be ruled out. Similar cases have been reported, including DLBCL later reclassified as TFHL-AI (11) and composite lymphomas with distinct DLBCL and TFHL-AI components (12). These highlight the risk of misdiagnosis when only one lineage is identified and emphasize the importance of evaluating TFH markers and T-cell clonality for accurate diagnosis and appropriate treatment. However, we acknowledge that T-cell clonality analysis was not performed on the initial biopsy, which represents a limitation in our evaluation.

PTCLs are aggressive malignancies with poor prognosis, low remission rates, and frequent relapses. Despite ongoing research, the outlook for TFHL-AI remains poor (13, 14). The 2022 NCCN guidelines recommends dose-adjusted EPOCH (etoposide, prednisone, vincristine, cyclophosphamide, and doxorubicin) as one of the first-line treatments, but its effectiveness is limited. Epigenetic drugs, which are currently investigated in clinical trials and recommended as second-line treatments (15), have shown potential for improving PTCLs outcomes (16). Chidamide, a selective HDAC inhibitor, has a unique mechanism of action. In a study of 383 patients with relapsed/refractory PTCLs, chidamide-based regimens showed encouraging results (17). Based on this, our patient was treated with chidamide plus COEP, which appeared to prolong survival. Notably, patients with TFHL-AI secondary to DLBCL typically survive only 2–3 months after TFHL-AI diagnosis (4, 18, 19).

We conducted a thorough literature review (**Table 1**) and found numerous reports of patients with TFHL-AI developing DLBCL, NOS after treatment (3, 20–26). In contrast, only three case reports describe the development of TFHL-AI following a prior diagnosis of DLBCL, and none included NGS analysis of tumor specimens before or after disease progression (4, 18, 19). These cases showed poor outcomes despite various treatments, highlighting the aggressive nature of such progression.

**Table 1:** Review of relevant literature on the interconversion between TFHL-AI and DLBCL, NOS.

Age	Gender	Disease 1& treatment	Disease 2 & treatment	Clinical outcomes	References
47	M	TFHL-AI, CHOP	EBV+DLBCL, NOS, R-GDP	remission	(3)
78	M	TFHL-AI, CHOP	EBV+DLBCL, NOS, lenalidomide & chidamide	dead	(20)
56	M	TFHL-AI, CHOP	EBV+DLBCL, NOS, PD-1 inhibitor & GDP regimen	PR	(21)
65	M	TFHL-AI, CHOP	DLBCL, NOS, CHOP-R	PR	(22)
68	M	TFHL-AI, CHOP	EBV DLBCL, NOS, COP-R	remission	(23)
36	F	TFHL-AI, FED & AHSCT	DLBCL, NOS, CHOP-R & ASCT	CR	(24)
68	F	TFHL-AI, CVP	EBV+ DLBCL, NOS, ICE	dead	(25)
64	M	TFHL-AI, IHOP	DLBCL, NOS, CHOP-R	PR	(26)
83	M	EBV+DLBCL, NOS, R‐miniCHOP	TFHL-AI, chidamide and prednisolon	OS 15 months	(4)
73	M	DLBCL, NOS, R-CHPVD	TFHL-AI, GLA	OS 8 months	(18)
72	F	DLBCL, NOS, BR	TFHL-AI, no treat	NA	(19)

M, male; F, female; TFHL-AI, TFH-cell lymphoma, angioimmunoblastic-type; CHOP, cyclophosphamide, adriamycin, vincristine, and prednisolone; EBV+DLBCL, NOS, EBV-positive diffuse large B-cell lymphoma, not otherwise specified; R-GDP, gemcitabine, dexamethasone, cisplatin, and rituximab; PR, partial remission; CHOP-R, cyclophosphamide, doxorubicin, vincristine, prednisone, and rituximab; COP-R, cyclophosphamide, doxorubicin, prednisone, and rituximab; FED, fludarabine, cyclophosphamide, and dexamethasone; AHSCT, autologous hematopoietic stem cell transplantation; ASCT, Allogeneic stem cell transplantation; CR, complete remission; CVP, cyclophosphamide, vincristine, and prednisolone; ICE, iphosphamide, carboplatin, and etoposide; IHOP, ifosfamide, doxorubicin, vincristine, and prednisone; R-CHPVD, rituximab, cyclophosphamide, pirarubicin, vindesine and dexamethasone; GLA, gemcitabine, lobaplatin, and L-asparaginase; OS, overall survival; BR, bendamustine and rituximab.

In summary, this case highlights a potential clonal relationship between DLBCL, NOS and TFHL-AI driven by shared *TET2* mutations. The poor prognosis and diagnostic challenges highlight the need for thorough evaluation. Chidamide-based chemotherapy may offer a potential treatment option.

## Data Availability

The original contributions presented in the study are included in the article/supplementary material. Further inquiries can be directed to the corresponding author.
